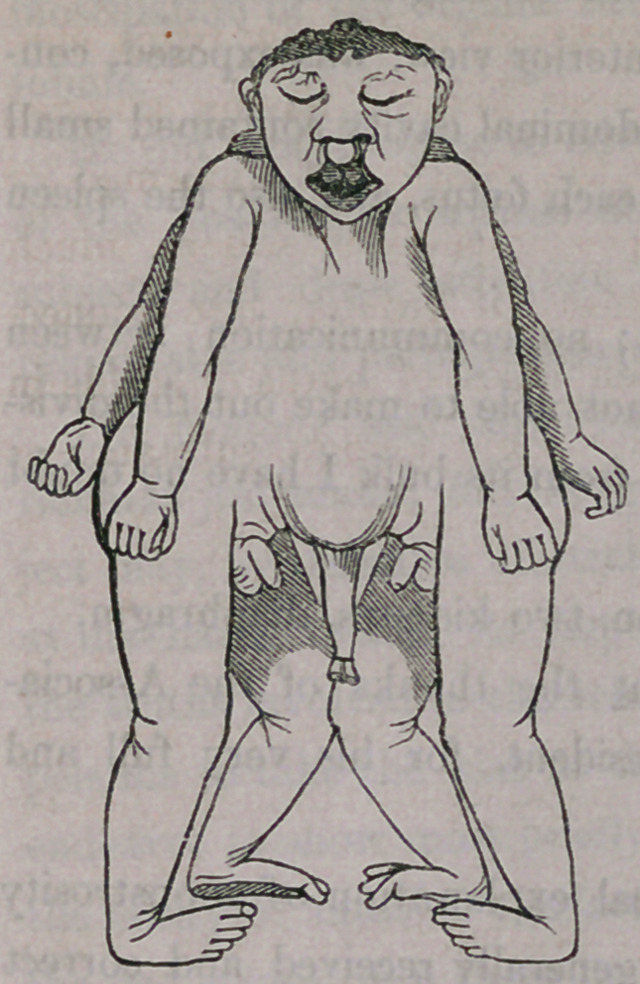# Abstract of the Proceedings of the Buffalo Medical Association

**Published:** 1862-11

**Authors:** J. F. Miner

**Affiliations:** Secretary


BUFFALO

### Medical and Surgical Journal

VOL. II. NOVEMBER, 1862. NO. 4.

### ORIGINAL COMMUNICATIONS.

ART. I.—*Abstract of the Proceedings of the Buffalo Medical Association.*

TUESDAY EVENING, October 7, 1862.

Present—Prof. James P. White, President, in the Chair; Drs. Rochester, Wyckoff, Shaw, Samo, Congar, Cronyn, Miner.

Minutes of the last meeting were read and accepted.

*J. B. Samo*, Treasurer, read his semi-annual report, showing a balance of accounts.

Voted that the report be accepted and placed on file.

*Prof. White* read the following letter:

HORNELLSVILLE, October 1st, 1862.

Prof. J. P. White:

Dear Sir:—I send you the following case of monstrosity, or united twins, which you are at liberty to communicate to the Buffalo Medical Association if you think proper.

I was called to attend Mrs. B. on Sunday the 21st ult., at 7 o'clock P. M., and found she had been in labor since one o'clock of that day. An examination showed the os-uteri well dilated, and the membranes protruding through it. The waters passed off, and the pains became feeble.—Stimulants and wine of ergot were given without any perceptible effect. She continued to have but little uterine effort until nine o'clock of the next day, when I again resorted to the use of ergot in large and frequent doses, but without altering the character of the uterine pains. At eleven o'clock I applied the forceps and readily delivered the head, which presented in the first position. I found great difficulty in bringing down the shoulders, but after delivering the one corresponding to the sacrum of the mother, I was totally unable to deliver the other by any force I judged prudent to apply.

Having previously placed my patient under the influence of chloroform, I passed my hand by the side of the head into the vagina, and much to my surprise found a confusion of arms and legs, which appeared to be attached to the body of the child whose head I had already delivered. On further examination I found what appeared to be a second child lying in the transverse diameter of the superior strait, but connected by some, (to me unaccountable manner at the time,) to the body of the child partly delivered. I passed my hand along the body and over the thighs of the second child, and found by bringing down the extremities I could, much to my satisfaction, terminate the labor with safety to the mother.

For a description of the children I refer you to the enclosed statement, which is substantially correct, being taken from notes dictated by me at the time.

The mother, aged twenty-eight, has had one child; suffered much during present pregnancy.

*The children*—external appearance. Heads perfect in all respects, and wholly separate; one measuring twelve, the other eleven inches around the parietal protuberances, and covered with hair. Necks also perfect and separate. Only one body from top of shoulders down to the umbilicus, measuring thirteen inches around the thorax. Two distinct, perfect arms at the sides of the body; a third, large arm, containing the bones of two arms, and terminated by two perfect, separate hands, with palms facing each other, on the back, opposite the sternum. One common cord, and one placenta. Two separate, perfect bodies, below the umbilicus. Legs and feet well formed, and separate. (Two perfect children from the umbilicus down—both females.) Length of children eighteen and one-half inches. Weight of the two seven and one-half pounds.

Dissection showed one common sternum, with corresponding ribs: two hearts, two sets of lungs, one liver, two gall ducts, and separate intestines to each.

*The Labor.*—Pains commenced on Sunday, September 22, 1862, at one P. M., and increased gradually in intensity until seven P. M., coming on after intervals of about ten minutes. Examination at seven P. M. showed the cervix well dilated, and the bag of waters protruding. Motion on the part of the fœtus was felt up to this time, but not afterwards. At about this time the pains became feebler. Stimulants, hot pepper tea, and diluted alcohol were given without increasing the pains. The presenting head was now at the bottom of the pelvic cavity. Wine of ergot was next given with no other effect than making the pains more continuous. Pains continued at intervals of three to five minutes until nine A. M. of the next day, (Monday.) Wine of ergot, in large and frequent doses, was again given without increasing the expulsive action of the womb. No change occurring up to eleven A. M. the forceps were applied to the presenting head, (which was the smaller,) and it was delivered, as the body and larger head lay directly across the superior strait. Great difficulty was also found in bringing the shoulders down. One of the shoulders was next brought into the world. The other not following as is customary, the hand was inserted by the side of the head and the mass of arms and legs was felt. The hand was passed over the body, and the fingers being curved into the bend of the thigh (at its upper extremity,) the legs of this child with the body were brought out, the larger head behind, which was without difficulty brought away.

The mother was not torn by the operation; rested well the first night; no unfavorable symptoms appearing; slept the whole of the second night, (only one Dover's powder with a little morphine having been given;) promises fair to be up in less than a week.

Respectfully yours,

C. C. Robinson.

*Dr. White* remarked, in presenting the foregoing communication, that it possessed considerable interest, as an illustration of the manner in which embryo occupying the same amniotic sac were sometimes united. It is now believed by most physiologists that "monsters" of the kind here described, are produced during the early stage of embryonic existence by the joining, or welding of two bodies together in such manner as to make but one possessing more or less of the organs of both. In twins contained in the same amniotic sac, the embryo or fœtuses having attained a certain size, are brought into co-aptation, the cuticle yet unformed or exceedingly vascular and imperfect, the opposing surfaces adhere, much as the labia majora may be made to agglutinate, if brought into connection with the cuticle removed. All have seen cases of burns or scalds in fingers, which, for want of care in interposing some foreign substance or dressings, during the process of healing, have united and left the fingers webbed. In the same manner do the exterior of the surfaces in utero unite or adhere. Happily when twins are conceived they inhabit, ordinarily, each its own amnion, and in some instances its own chorion, which insulates them, and they float in the liquor amnii and remain separate.

Again, the different sides or parieties of the embryonic body unite, both anteriorly and posteriorly, in the middle line, hence we perceive that the opposite sides of the two fœtal trunks might be brought in contact, and thus unite the bodies of different children at this central line. Thus the right side of one would adhere to the left side of its neighbor.

A remarkable case, bearing upon this subject, and which illustrates the position assumed of the mode of production of the deformity in this class of cases, that the opposite halves of bodies united most readily, is often referred to by modern writers, and which was first described by M. Serres. This monster was born in the kingdom of Sardinia, in the year 1829.—There were two heads, a double thorax, with four arms, and one abdomen, with two legs only. They lived to the age of eighteen months, and were christened, the one Rita, the other Christina. When these children were asleep, and the right foot was tickled, Rita would wake and smile; so if the left foot was tickled, Christina was made to laugh, without at all affecting her sister, for this was Christina's leg, and not Rita's, and vice versa. The individuality or duality of bodies similarly constituted might form a curious subject for reflection. Some specimens have one head and two bodies, whilst others have two heads and one body. Some have two sets of superior extremities and one only of inferior; whilst with a double set of inferior extremities and two complete sets of genital organs there is sometimes but one head.

Here is a specimen deposited by himself in the museum of the University of Buffalo, which combines in itself a large number of departures from the natural standard in its external conformation. It weighed ten pounds and was without difficulty delivered from a healthy mother, who had previously been confined with three well developed children. This monster has one head only, which is acephalous, thus illustrating another form of monstrosity from the absence of parts. It has double hair-lip, and split or double tongue. There is but one neck, one body to the hips, two sets of superior and two sets of inferior extremities, having between them two complete sets of male genital organs. One aspect of the body is abdominal, having an umbilicus to which was attached the single funis; whilst upon the dorsal aspect there are two spinal columns which are perfect. This monster died soon after birth, as indeed is the case with all acephalous formations, being incapable of independent existence involving respiration, &c., &c.

The case described in the contribution furnished by Dr. Robinson, differs from any with which I am familiar in the third superior extremity, or double arms with two hands. The bodies seem here to have united, taking the half of each, whilst the heads and extremities and genitalia remain separate, except the enclosure of the bones of two arms in the same integument, the hands still being perfect and separate.

Intending to be very brief, without referring to any of the other forms or varieties of monstrosity or to their cause, he would submit the account of this case by Dr. Robinson to the consideration of the members of the Society.

The following minutes of dissection were furnished by Prof. Wm. Mason: Upon cutting through anterior abdominal surface and cartilaginous sternum, the thoracic and abdominal cavities were exposed. The former contained heart $1\frac{1}{2}$ inches in length, and lungs. The latter, a large liver, 3 inches square, filling the whole cavity anteriorly; two kidneys and spleen Of these, the heart, lungs, liver and kidney at left side, belonged to the fœtus upon left side, while the spleen and kidney at right side appertained to the fœtus upon right side.

Upon cutting through posterior surface and cartilaginous sternum, the thoracic cavity of the fœtus at right side, in anterior view, was exposed, containing a rudimentary heart and lungs. Abdominal cavity contained small liver, two kidneys, one of which belonged to each fœtus, and also the spleen of the fœtus upon left side in anterior view.

The two thoracic cavities were distinct; no communication between them. Owing to condition of parts I was not able to make out the divisions of the alimentary canal, though judging from its bulk I have no doubt of its being double.

Summary—Two hearts, lungs, livers, spleen, two kidneys, diaphragm.

On motion of Prof. Rochester, *Voted* that the thanks of the Association be presented Dr. Robinson by the President, for his very full and interesting report.

*Dr. Miner* remarked that the physiological explanation of monstrosity referred to by Prof. White, was perhaps the generally received and correct theory concerning it, and that many cases might be satisfactorily accounted for in this way, while other cases might not so well illustrate the correctness of the observation. When children are joined together, or where perfectly formed parts are irregularly attached, and even where one fœtus is enclosed in another, it may be accounted for very plausibly by the welding of parts, or the union which takes place at a certain period of development; while misshapen parts or supernumerary organs are not so fully explained by the theory. That mental impressions made upon a pregnant woman may be, and sometimes are, conveyed to the fœtus in utero, he was unwilling wholly to ignore. Had seen some cases and been well acquainted with the facts which served to illustrate this position, with great force.—Mentioned a case where the imitation of a pig's nose was placed upon a child's face, and said that many remarkable instances are well authenticated where impressions made upon the mind of the mother seem to be communicated to the fœtus. In saying this he did not propose to disprove any observations which have been made accounting for monstrous formations, or intimate any deficiency or incorrectness, but simply suggest that the welding process or other processes may sometimes be influenced by mental emotion. Since Prof. White had avoided all reference to this subject, and cheated us of his opinions, which we should have regarded almost conclusive, he would say that Dr. O'Reilly in his recent work upon the Placenta and Nervous System, had not only attempted to show that these impressions are made, but *how* that they are conveyed through the inosculation of the organic nerves of the mother and fœtus in the placental lobule.

He did not propose to advocate for O'Reilly's views, attempt any proof of the position, or express any personal opinions, but to speak upon the subject and draw attention to the old fashion way of explaining these remarkable and perhaps inexplicable freaks in nature.

*Dr. Congar* said, Mr. President, I rise hesitatingly in response to the remarks just made; for, being no orator, I greatly fear this interesting subject may, by defective illustration, fail of receiving the appropriate advocacy at my hands, which its importance deserves; and yet, my attachment to the truths involved in the relations to which reference has been made, compels me to take the risk of doing injustice by default to this subject in an endeavor to show most briefly the great probability, if not the certainty, of the falsity of most of the facts, and the crudity, and even the absurdity of the deductions from them by the advocates of these notions, both among the people and in the profession. I feel myself compelled to regard all the attested maternal influences on the *fœtus in utero*, beyond that which the *laws of hereditary descent* assign to the mother at the time of conception; and, beyond that which affects the *health* of the fœtus through the varying characteristics of the nutritive material furnished by the mother through her intimate organic connection with the offspring, as mere fancy; and fancy based also on false facts; and the whole uncorrected by any adequate knowledge of the true laws of the hereditary influence of parents on offspring, or even of the existing relations of the physiological functions which are under the control of these laws. For the physiological reasoning of Prof. Wm. P. Dewees, in the beginning of the present century, has always appeared to me conclusive so far as it went; and I consider it as good now as then, and therefore as immutable as physiological laws. He states, (and the statement has never to my knowledge been disproved,) that there is no *nervous connection* discoverable between the depurative organ of the fœtal blood, the placenta, and the uterus of the mother. That, inasmuch as *nervous connection* with the nervous centres of the brain and spiral cord, in a healthy organism, essential to *any* and *all physical influence to, and over* any and every part of such organism, the *absence* of such nervous connection between the fœtal organization and the nervous system and centers of the mother, forbid the expectation and even the supposition of any direct physical influence from, or through the mother over the fœtal organization after conception. But were these physiological deductions inconclusive, I here in my place declare, that to my mind, the suppositions (for they are nothing more,) which I oppose, are inconsistent with all the principles of the hereditary descent of character from parents to offspring; for, if, according to its primary general law, "*children are daguerreotypes of parental conditions at the time of conception*," the popular doctrine of maternal influence cannot be true; because, according to it *external influence*, through the mind and nervous system of the mother, can mar and modify this likeness to any extent; yes, it can even destroy it. Now, if this is true, (and who will deny it?) it must also be true of the several parts of which the general law is composed; that is, the direct and indirect paternal, and the direct maternal influence in the formation of the innate character of offspring, both organic and physical, are alike at the mercy of the external world, through the mother, for integrity, balance, and even existence! The mere statement of the case from this stand point shows the absurdity and falsity of the popular notion. And now, in all candor we ask, is not this notion of the production of marks, monsters, &c., &c., by maternal impressions from without, based entirely on a false interpretation of the ethnological facts from which science derives the evidence of the existence of man's secondary, his indirect influence through the mind of woman in the formation of the innate character of offspring? If this is so, can we be wrong in the assertion that this extravagant extension, and erroneous application of maternal influence, owe their existence entirely to an ignorance of what the true laws of hereditary descent are? and, of the right direction and real extent of their respective relations in the race?

*Dr. Cronyn* said he had bestowed some thought upon the subject which had been thoroughly investigated in England and France, the discussions extending over a period of several years. He thought it settled that these developments were due to physiological processes, uninfluenced by mental emotions. Spoke also of the seminal fluid upon the side of an egg being often streaked with blood, and suggested that it might have some influence in producing a mark upon the chick.

*Dr. White* remarked that he should have said, that Dr. Congar had furnished him the best specimen of a fœtus in the process of welding or joining, he had yet seen, the process having been arrested while the union was yet incomplete.

*Dr. Rochester* recalled to the memory of the members of the Association, the interesting report of a case of pelvic hæmatocele, recently made by Prof. White, as he had a contribution of a somewhat similar character to offer. He visited on the sixth day of August, a woman who supposed she was ill from the effects of an early abortion; she was a prostitute, thirty-four years of age, and had not been pregnant for eighteen years. Late in July, after protracted and violent coition, in a most unnatural attitude, she found herself unable to urinate. In the course of a few hours she was seized with violent uterine hæmorrhage, attended with constant hypogastric pain, and paroxysms of tenesemus and strangury. She was attended by a medical gentleman of the City, who treated her very judiciously, but finally discontinued his visits. When Dr. Rochester was called he found that the hæmorrhage had nearly ceased, but the hypogastric pain was constant, and there was entire inability to evacuate either the bladder or the rectum; there was, moreover, always present a sensation of great distension in the lower pelvic region. There was no evidence that abortion had taken place, except the hæmorrhage. The case was regarded as one of metro-cystitis, but no vaginal examination was made on account of intra-vulval chancres of forbidding aspect. No improvement having taken place for ten days, the risk of infection was hazarded, and the touch detected a prominent, tender and painful tumor in the upper part of the posterior vaginal wall. The os-uteri was crowded up behind the pubic arch and by Simpson's sound the uterus was found retroverted and elongated the fundus was carried up by the sound, but the vaginal tumor remained. Examination by the rectum, which caused agonizing pain, and which was attended with difficulty also detected a large, firm mass in the septum.—It was evident that there was present a tumor; was it fibrous, or was it a dislocated ovary, or a collection of pus or blood? Probably one of the two latter. On the day succeeding this discovery, Prof. White, in consultation, expressed the opinion, that from the peculiar elastic sensation or palpation, pus would be found, and passing, by request, an exploring needle, he demonstrated the soundness of his conjecture. On the same afternoon Dr. Rochester made a free incision with a large bistoury, and more than a pint of most offensive pus was immediately discharged. Simpson's sound was carried up seven inches, showing the depth of the abscess. Although the incision was large and cruciform, it was difficult to keep the opening from healing, and it was found that a large tent or bougie must be worn to keep it patulous—large injections of warm water constituted the rest of the local treatment. Quinine, iron, opium and stimulants were given freely for four weeks, and now at the end of two months, the abscess has healed, and the patient has (probably) resumed her avocation.

Dr. Rochester stated that he preferred to make the incision through the vagina rather than the rectum, on account of the facility afforded by the former for local treatment, and also because he thought that an opening through the rectum might be blocked up by fæcal matter, or that the fæces might even pass into the bed of the abscess.

Dr. Rochester called attention to the uterine hæmorrhage, coincident with the formation of this *abscess*, as he had a case of pelvic hæmatocele now two days under his treatment, on which he hoped to report at the next meeting, the formation of which was attended with or preceded by uterine hæmorrhage of two week's duration. He was inclined to believe that these recto-vaginal exudations were the result of direct violence or straining, and that accident or the peculiar condition of the patient caused sometimes pyocele and somotimes hæmatocele, and that their pathology was primarily not essentially diverse.

*Dr. Miner* had recently attended a widow lady in the city, aged forty-five years, who after complaining for a week of some uneasiness in the pelvis, was seized suddenly with violent pain in the lower part of abdomen, while at the market for vegetables; she walked home, the pain continuing very severe, and soon accompanied by protracted chill. Opiates were prescribed and warm fomentations applied, when the severe pain considerably abated. For three or four days she seemed to be improving in many respects, but complained of great fullness in the pelvis, and constant desire to urinate and have passage from the bowels. Upon examination per vagina the os-uteri was high up above reach, and the tumor or accumulation seemed to fill the pelvis completely. Upon rectal examination the accumulation seemed equally extensive, and passed backwards, filling the hollow of the sacrum. Without any hesitation, and indeed without estimating any dangers of mistake, since his attention had not then been called particularly to that form of disease, he introduced through the rectum a large sized trocar and canula, and drew off a common sized bed vessel at least half full of the most intolerably offensive pus he had ever found in any location. The idea of its being anything but pus did not enter his mind since the chill, fever, pain, and all the symptoms which he could not previously explain, seemed to be fully accounted for upon the discovery of the abscess. In regard to the diagnosis, he claimed no credit for his discovery, and only wondered at his stupidity in not finding and opening it sooner. He used a canula for the purpose of cleanliness, expecting to be thus able to convey the pus to the vessel, but should have had no hesitancy in opening it with scalpel, lancet, or other instrument by which opening could be made sufficiently large. Selected the rectum in preference to the vagina, with the view that any subsequent discharge would be retained in the rectum, and only voided when necessary. No further attentions were required in the case; with each discharge from the bowels was large quantities of pus, and no tendency to closure of the opening or re-accumulation was manifest. The evacuation of the bladder and bowels became natural, and all symptoms of the disease disappeared, the woman very soon regaining her usual health and strength.

*Dr. Cronyn* related a similar case in a married woman, whose husband was not at home, but he could not say how it was about sexual intercourse acting as a cause. She complained of severe bearing down pains. The uterus was retroverted. Passed into the uterus a uterine sound, and Dr. Wyckoff, who visited the woman with him, held the instrument while he introduced his finger into the rectum to explore more fully the tumor; while thus manipulating, the rectum gave way, giving exit to a large quantity of pus; the odor was awful. Patient soon recovered, and is well.

*Dr. Wyckoff* mentioned a case in his practice where Dr. White, while in consultation, passed an exploring trocar without results. It afterwards opened spontaneously into the rectum, and was followed by rapid recovery.

*Dr. White* remarked that in these cases we have not to diagnose whether it is blood or pus, or fundus of uterus only; other tumors might occupy this position. Dr. Samo had invited him to see a case where the indications were that it was pelvic hæmatocele or cellulitis. Dr. Samo gave chloroform and he introduced an exploring trocar, then a larger, and afterwards a still larger, when no blood escaped, and they had to leave it and acknowledge the operation incomplete. It was probably ovarian or extra mural. He had also another patient who seemed to have a similar condition, but did not; so we must not come to the conclusion that it is either pus or blood. In the case of Dr. Samo's it seemed certain to be one or the other, but proved to be otherwise.

*Dr. Cronyn* said that Mr. Hilton in his lectures upon conservative surgery says, nature often points to the proper place for operation. He also recommends the lancet to any other instrument for opening such accumulations. Nature seems to point to the rectum; both his own and Dr. Wyckoff's case, with numerous others, would illustrate that nature chooses the rectum.

*Dr. White* replied that he thought nature quite as often chose the vagina; that they were quite as apt to open into the vagina.

*Dr. White* would mention a case of hæmatamesis. A young farmer, twenty-eight years old, healthy and vigorous, had been engaged haying. Sunday evening while milking he vomited two quarts of blood. Monday about the same quantity. Wednesday and Thursday each day, about another quart; the ordinary results of so great loss of blood were produced. Dr. Potter, who was in attendance, had used all the common remedies in such cases. Dr. White suggested solution per sulphate of iron, since which time he has had no hemorrhage. It was remarkable that so healthy a young man should have so severe hemorrhage from the stomach. Its suppression seemed to be due to the iron, still it might have been mere coincidence.

*Dr. Cronyn* related a case of similar character. After trying various remedies it was controlled by five grain doses, every two hours, of perchloride of iron; no more hemorrhage after the second day. There was present great tenderness of the stomach, as if from active congestion. The patient rallied and was soon well.

*Dr. Rochester* had used per sulphate of iron in menorrhagia; thinks it does not constipate the bowels, but perhaps acts also as a tonic. Has used it also in other affections, but not in hæmatamesis.

*Dr. Wyckoff* had recently been called to see a young child with fractured clavicle. It had received no fall or injury. Inquired if fracture of the clavicle could occur during labor, or be produced by it; there was no other known mode of inquiry.

*Dr. Wyckoff* also mentioned a well marked case of cholera, which he had treated recently; it was as clear a case of cholera as he had ever seen. The ejections from the stomach were of a peculiar briney fluid, while the discharges were rice water. The case occurred on Mechanic street.

Voted to adjourn to the first Tuesday evening in November.

J. F. Miner, Secretary.

## Figures and Tables

**Figure f1:**
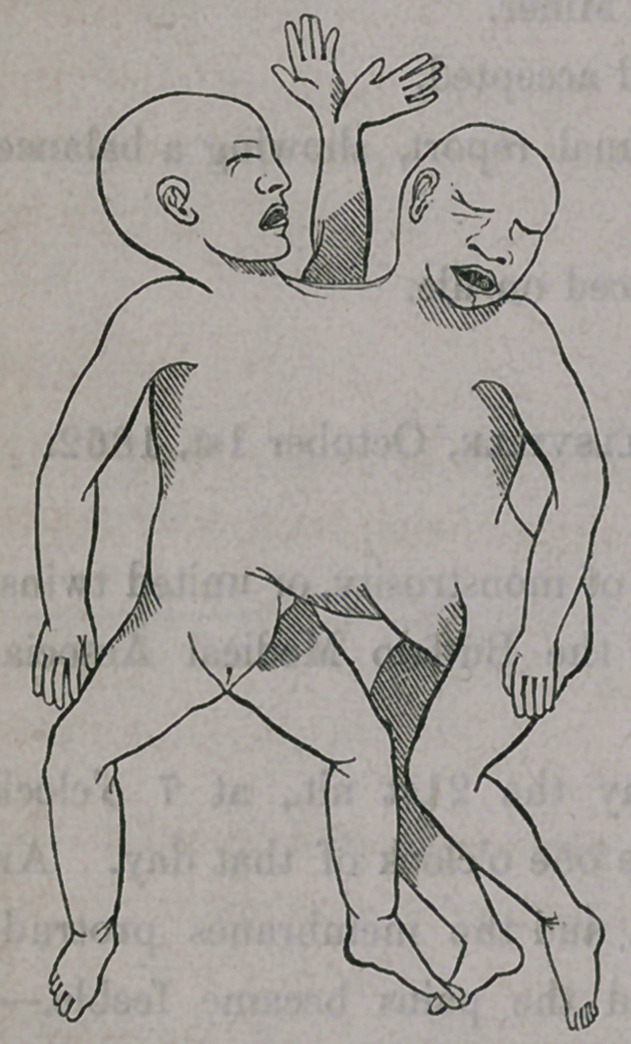


**Figure f2:**